# Supplementary material for: Single molecule analysis reveals monomeric XPA bends DNA and undergoes episodic linear diffusion during damage search
Source: Nat Commun. 2020 Mar 13;11:1356. doi: 10.1038/s41467-020-15168-1 (PMC7069974; doi:10.1038/s41467-020-15168-1)
Supplement: Supplementary file 1 — Supplementary Information [file 41467_2020_15168_MOESM1_ESM.pdf]

SUPPLEMENTARY INFORMATION

Single molecule analysis reveals monomeric XPA bends DNA and undergoes episodic linear diffusion during damage search

Beckwitt et al.

Supplementary Note 1. Estimating specificity from AFM binding position. As published by Erie and colleagues, a Gaussian model with an additional term accounting for non-specific binding can be fit to position data obtained from AFM experiments and used to assess specificity without confounding end-binders[1]. Specificity calculations from protein binding positions were performed as published[1]. A Histogram showing the distribution of protein binding position between 0 and 50% contour length of AAF_{538} was fit by least squares nonlinear regression: $y = 3.6 + 10.4e^{-0.5\left(\frac{x-34.3}{9}\right)^2}$. Specificity ($S$) was calculated as:

$$S = N * \frac{A_{specific}}{A_{non-specific}} + 1 \qquad \text{Equation 6}$$

where N is the number of potential binding sites using an estimate of 8 bp (ref. [2]) for the DNA footprint of XPA (N = 538 bp – 8 bp + 1 = 534 bp) and $A_{specific}$ and $A_{non-specific}$ are the areas under the curve representing specific and non-specific binding, respectively (Supplementary Fig. 2). Using this method, we obtain a specificity factor of 660 for the dG-C8-AAF site.

Supplementary Note 2. Validation of methods to determine the AFM volume of a protein bound to DNA. It was important to validate that the standard curve based on free proteins (Fig. 2b) also permitted the analysis of the volumes of DNA-bound proteins. We therefore validated our protocol (see Methods and Supplementary Fig. 4a) using two proteins of similar size and known stoichiometry. APE1 (37 kDa) was incubated with a 514 bp DNA substrate containing a nick at 36% from one end ($Nick_{514}$) and imaged by AFM (Supplementary Fig. 4b). The distribution of AFM volumes for APE1 on the DNA was centered at 40.6 ± 10.7 nm^3 (Supplementary Fig. 4c). This is very close to the AFM volume obtained for the free protein (40.4 nm^3, Supplementary Fig. 3b). Furthermore, using the standard curve, this corresponds to a molecular weight of 37.4 ± 11.2 kDa. Using our methods for DNA volume subtraction combined with the standard of free proteins, we were able to accurately estimate the molecular weight of APE1.

This analysis was also pursued with Polβ (42.8 kDa). Again, the protein was incubated with $Nick_{514}$ and imaged by AFM (Supplementary Fig. 4d). The distribution of AFM volumes was centered at 31.9 ± 16.1 nm^3, which corresponds to 29.8 ± 15.9 kDa (Supplementary Fig. 4e) and was smaller than expected. The AFM volume of the free protein was 50.7 nm^3 (Supplementary Fig. 3c), suggesting that, in this case, the contribution of the DNA to the total complex volume was over-estimated. This is a very likely explanation if the crystal structures of APE1 and Polβ are taken into consideration (insets, Supplementary Fig. 4c,e). APE1 is positioned on top of the DNA such that the volume of the complex is essentially the sum of the protein and the DNA alone, as our model assumes. However, Polβ pulls apart the nicked DNA backbone and inserts itself much further into the helix[3]; in this case, the volume of the complex appears to be less than the sum of the two parts. Overall, we can conclude that both

APE1 and Polβ bind DNA as a monomer using our method, but it is important to note that the accuracy of molecular weight estimates is dependent on the precise conformation of the protein-DNA complex.

Supplementary Note 3. Calculation of theoretical diffusion constants. Calculations to determine the theoretical limit of the diffusion coefficient and the energy barriers to free diffusion were pursued as described[4,5]. All calculations were done based on the streptavidin-coated 705 Qdot labeling strategy, although similar results are obtained for 605 Qdot conjugated to a secondary antibody. First, the hydrodynamic radii of full-length human XPA (3.3 nm, ref. [6]) and the streptavidin-coated 705 Qdot (12.8 nm, ref. [7]) were used to estimate the hydrodynamic radius of Qdot-labeled XPA (R_{eff} = 12.873 nm). Treating the labeled protein as a sphere allows us to define the diffusion coefficient (D) with the Stokes-Einstein equation:

$$D = \frac{k_B T}{\xi} \qquad \text{Equation 7}$$

where k_B is the Boltzmann constant (1.38 J/K), T is the temperature (298 K), and ξ is a friction term. The friction term for a protein sliding along DNA following the corkscrew path of the helix, described by Schurr[8] and modified slightly[9], is defined as:

$$\xi = 6\pi\eta R_{eff} + \left(\frac{2\pi}{10.5 \times BP}\right)^2 \left(8\pi\eta {R_{eff}}^3 + 6\pi\eta R_{OC} {R_{eff}}^2\right) \qquad \text{Equation 8}$$

where η is the viscosity of the medium (0.89 × 10^{-2} poise), BP is the distance between two DNA base pairs (0.34 nm), and R_{OC} is the off-center distance from the protein center of mass to the DNA helical axis (R_{eff} + 1 nm = 13.873 nm). Combining Equations 7 and 8 permits the calculation of the diffusion coefficient of Qdot-labeled XPA sliding along DNA with no energy barrier, or the theoretical limit to the diffusion coefficient (D_{lim}):

$$D_{lim} = \frac{k_B T}{6\pi\eta R_{eff} + \left(\frac{2\pi}{10.5 \times BP}\right)^2 \left(8\pi\eta {R_{eff}}^3 + 6\pi\eta R_{OC} {R_{eff}}^2\right)} \qquad \text{Equation 9}$$

Using the variables defined above for Qdot-labeled XPA, D_{lim} = 1.54 × 10^{-2} μm^2/s.

The energy barriers to free diffusion (E_A) can be calculated using the Arrhenius relationship:

$$k = e^{-E_A / k_B T} \qquad \text{Equation 10}$$

where k is the rate constant (in this case, the stepping rate $2D/BP^2$) and E_A is the activation energy of the reaction. The energy barrier to free diffusion is the difference between the theoretical (“barrier-less”) E_A and the experimentally determined E_A. By rearranging Equation 10 and substituting for k, this difference can be calculated:

$$\Delta E_A = ln\left(\frac{D_{lim}}{D_{expt}}\right) \times k_B T \qquad \text{Equation 11}$$

where D_{expt} is the experimentally determined D. Because the ΔE_A can also be used to describe the roughness of the energy landscape, this value may also be referred to as σ (ref. [10]). XPA undergoing

short-range linear diffusion had a mean D of 2.49×10^{-3} μm^2/s (Fig. 6b), thus permitting the calculation of the energy barrier to diffusion via Equation 11, $\Delta E_A = 1.57 \times k_B T$. The diffusion coefficient for XPA in the long-range mode (3.67×10^{-2} μm^2/s) exceeds the theoretical limit of a protein diffusing along the contour of DNA (Fig. 6b), so the relevant energy barrier could not be calculated.

Supplementary Figures

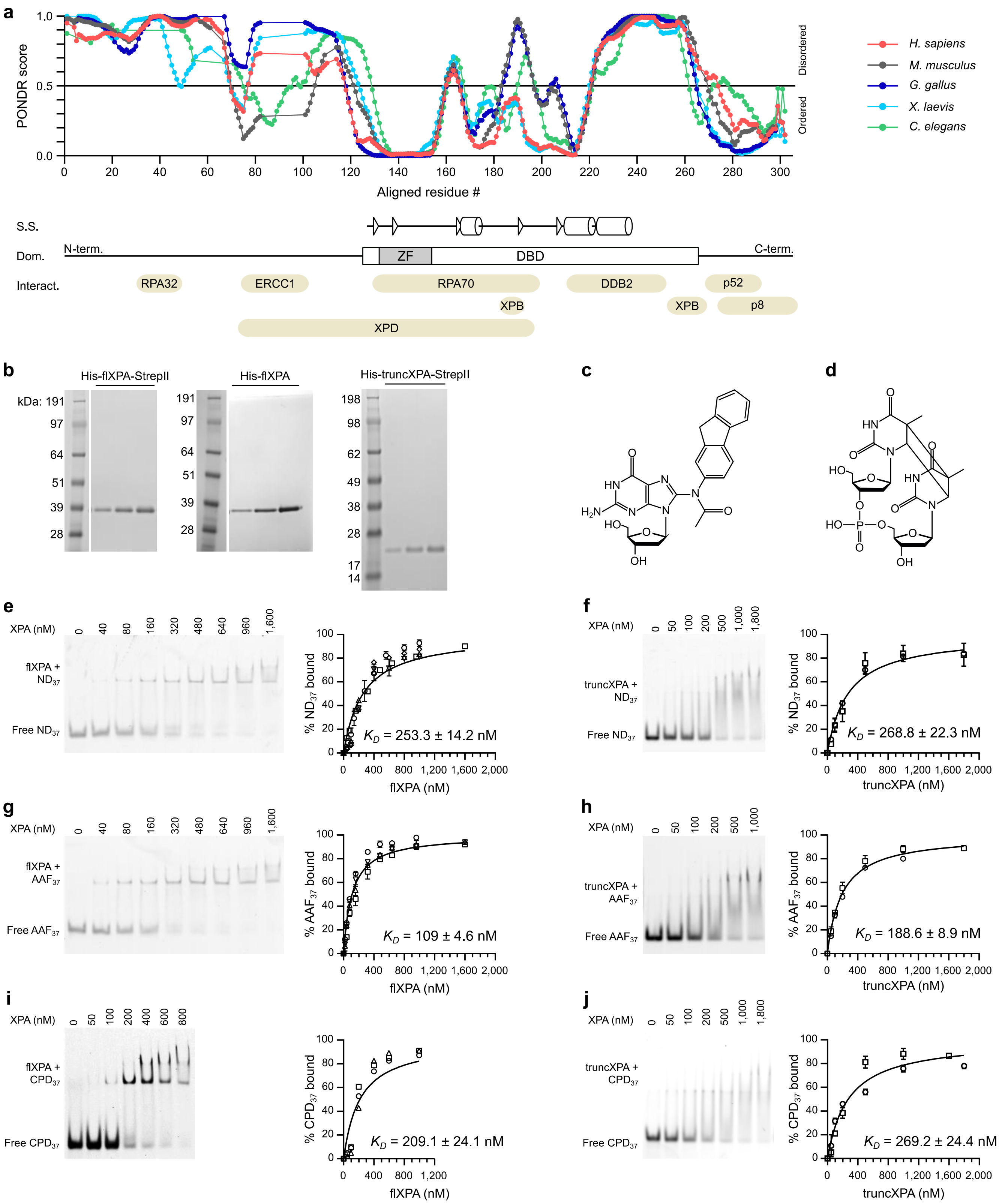

Supplementary Figure 1. Purification and DNA binding activity of full-length human XPA. a, Protein sequences of XPA homologs from multiple species were aligned using PROMALS3D[11] and disorder predictions for each sequence were obtained via the PONDR VL-XT algorithm[12]. S.S., secondary structure elements (triangles, beta sheets; cylinders, alpha helices) based on the NMR structure of the human XPA DBD (PDB 1XPA). Dom., conserved domains of the human XPA protein (ZF, zinc finger; DBD, DNA binding domain). Interact., some published interactions between XPA and NER proteins[13]; tan ovals represent XPA residues involved in the interaction. **b,** SDS-PAGE and Coomassie stain of purified His-flXPA-StrepII (loading amounts: 270 ng, 540 ng, 1.08 µg), His-flXPA (160 ng, 490 ng, 1.14 µg), and His-truncXPA-StrepII (110 ng, 230 ng, 340 ng). Ladders shown from the same gel with irrelevant lanes cut out. **c,** Chemical structure of N-(2'-deoxyguanosin-8-yl)-2-acetylaminofluorene (dG-C8-AAF). **d,** Chemical structure of a cyclobutane pyrimidine dimer (CPD). **e,** Left, representative EMSA gel showing flXPA binding to a 37 bp DNA substrate with a 5' fluorescein label (ND_{37}). Right, quantification of five experimental repeats (each run on duplicate gels) plotted as mean ± range of duplicate gels. The equilibrium dissociation constant (K_D) was determined by a global fit to the data (see Methods for model) and is reported as best fit value ± s.e. of the fit. **f,** Left, representative EMSA gel showing truncXPA binding to ND_{37}. Right, quantification of two experimental repeats plotted/fit as in e. **g,** Left, representative EMSA gel showing flXPA binding to a 37 bp DNA substrate with a central dG-C8-AAF adduct and a 5' fluorescein label (AAF_{37}). Right, quantification of four experimental repeats plotted/fit as in e. **h,** Left, representative EMSA gel showing truncXPA binding to AAF_{37}. Right, quantification of two experimental repeats plotted/fit as in e. **i,** Left, representative EMSA gel showing flXPA binding to a 37 bp DNA substrate with a central CPD lesion and a 5' fluorescein label (CPD_{37}). Right, quantification of three experimental repeats plotted/fit as in e. **j,** Left, representative EMSA gel showing truncXPA binding to CPD_{37}. Right, quantification of two experimental repeats plotted/fit as in e. Source data are provided as a Source Data file.

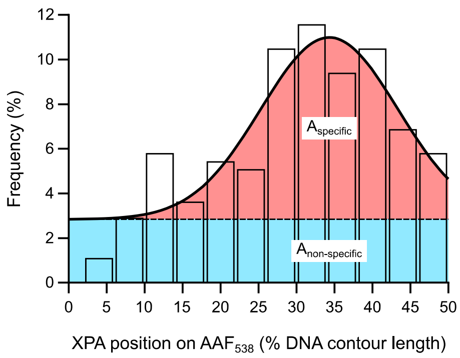

Supplementary Figure 2. Specificity analysis of XPA binding position by AFM. Histogram showing distribution of internally bound XPA (n = 217 particles, data reproduced from Figure 1e) on AAF_{538}. To calculate specificity, a Gaussian model with an additional term for non-specific binding was fit to the data. Red ($A_{specific}$), area under the curve representing specific binding. Blue ($A_{non\text{-}specific}$), area under the curve representing non-specific binding. See Supplementary Note 1 for calculation details.

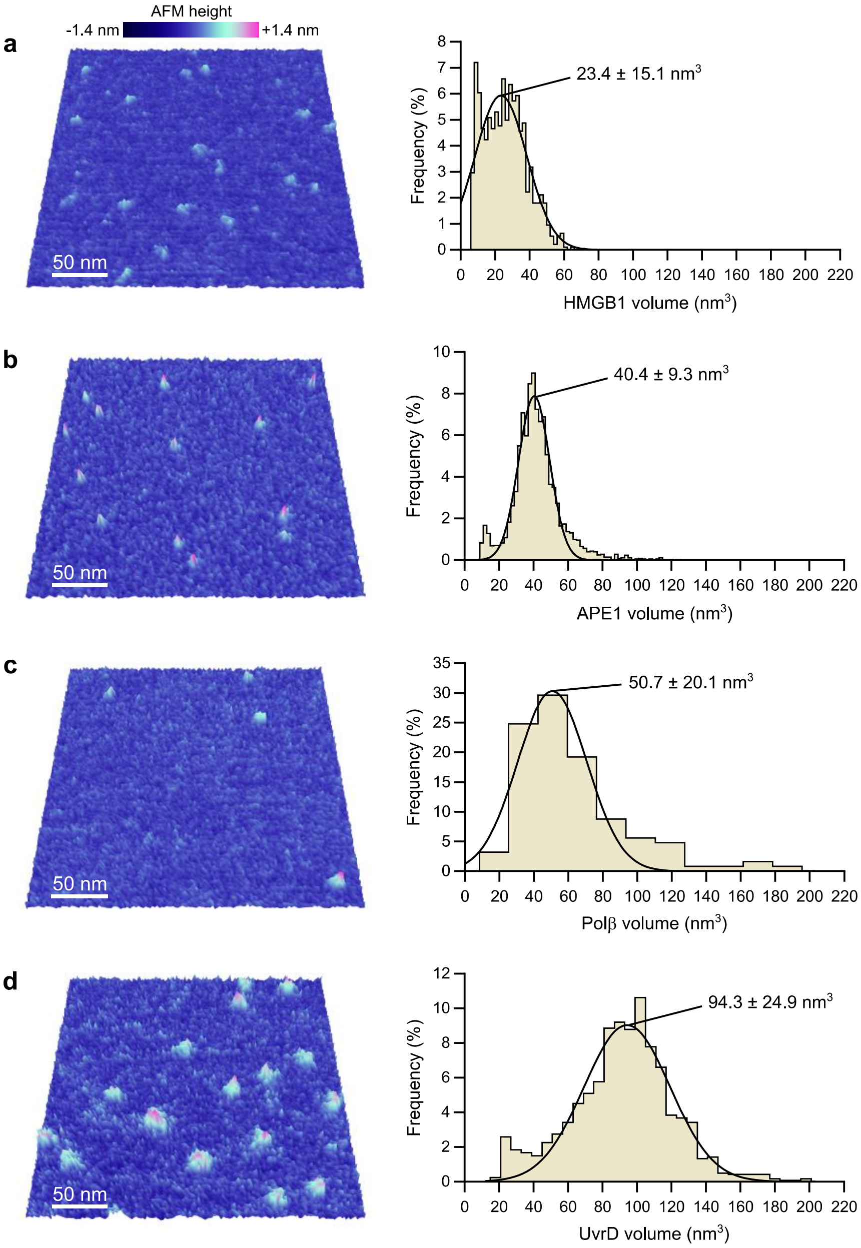

Supplementary Figure 3. Generation of standard for AFM volumes. a-d, Left, representative 3D AFM images and right, histogram and Gaussian fitting of AFM volumes used to generate the standard curve shown in Figure 2b. Color scale represents AFM height and applies to all panels. Gaussians are labeled with mean ± s.d. **a,** HMGB1, 25 kDa. n = 943. **b,** APE1, 37 kDa. n = 3,529. **c,** Polβ, 42.8 kDa. n = 125. **d,** UvrD, 85.6 kDa. n = 1,195.

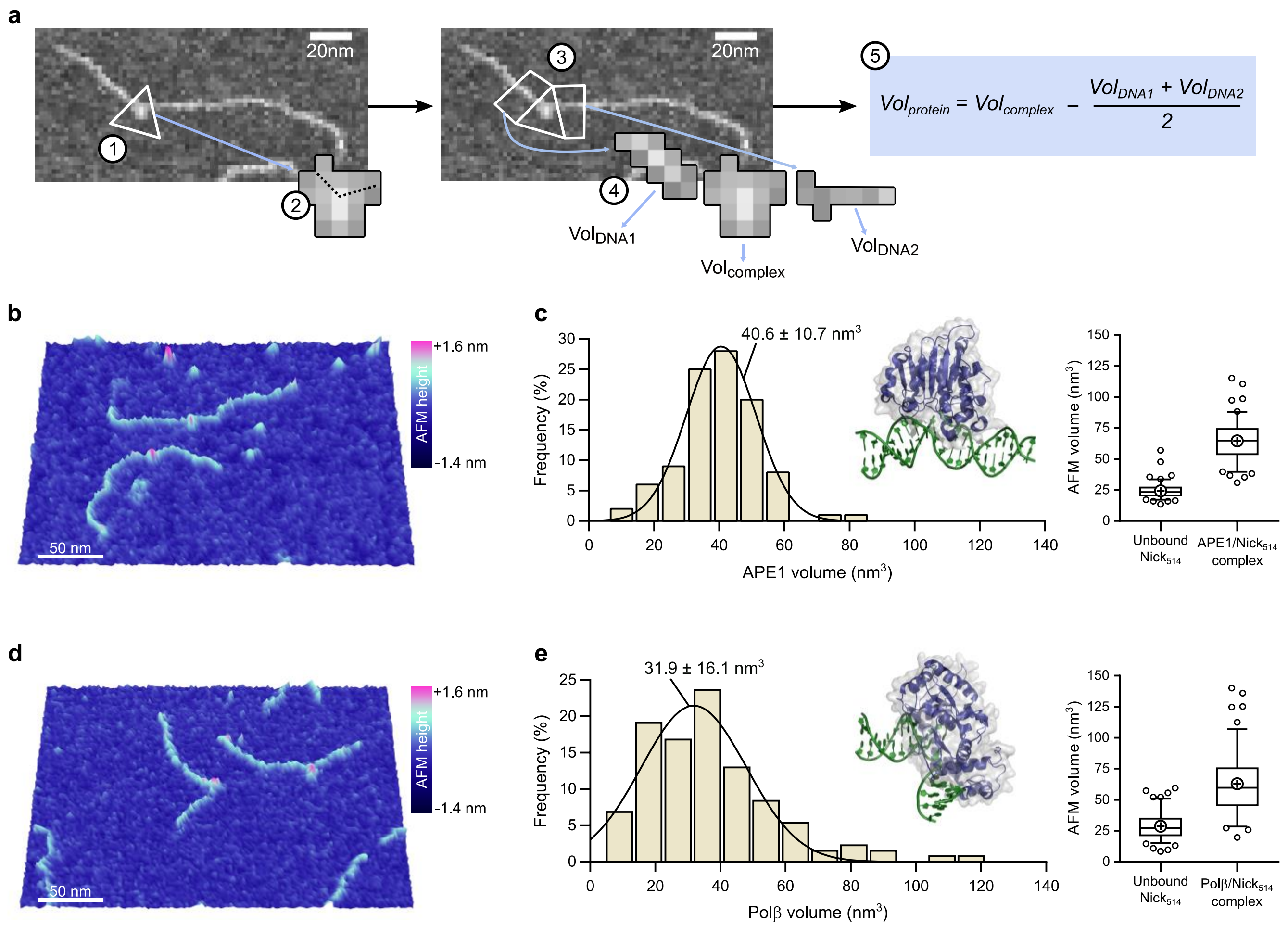

Supplementary Figure 4. Determination of DNA-bound protein AFM volumes. a, Schematic showing the steps used to subtract the DNA volume from a protein-DNA complex. (1) Outline perimeter of the complex to separate it from unbound DNA. (2) Measure contour length of the DNA path through the complex. (3) Delineate regions of unbound DNA on either side of the complex with the same length measured in step 2. (4) Obtain AFM volumes for all three regions. (5) Protein volume is calculated as the volume of the complex minus the average DNA volume. **b,** Representative 3D AFM image of APE1 bound to a 514 bp DNA substrate with a nick at 36% from one end ($Nick_{514}$). **c,** Left, histogram and Gaussian fitting of the distribution of calculated AFM volumes of APE1 on $Nick_{514}$ (n = 100). The Gaussian is labeled as mean ± s.d. The AFM volume corresponds to 37.4 ± 11.2 kDa. Inset, crystal structure of APE1 bound to a nicked abasic DNA substrate (PDB 5DFF). Right, box and whisker plots (5-95 percentile) of unbound DNA and total complex volume measurements. **d,** Representative 3D AFM image of Polβ bound to $Nick_{514}$. **e,** Left, histogram and Gaussian fitting of the distribution of calculated AFM volumes (n = 131). The Gaussian is labeled as mean ± s.d. The AFM volume corresponds to 29.8 ± 15.9 kDa. Inset, crystal structure of Polβ bound to nicked DNA (PDF 1BPZ). Please see Supplementary Note 2 for discussion of the underestimation of the protein size. Right, box and whisker plots (5-95 percentile) of unbound DNA and total complex volume measurements.

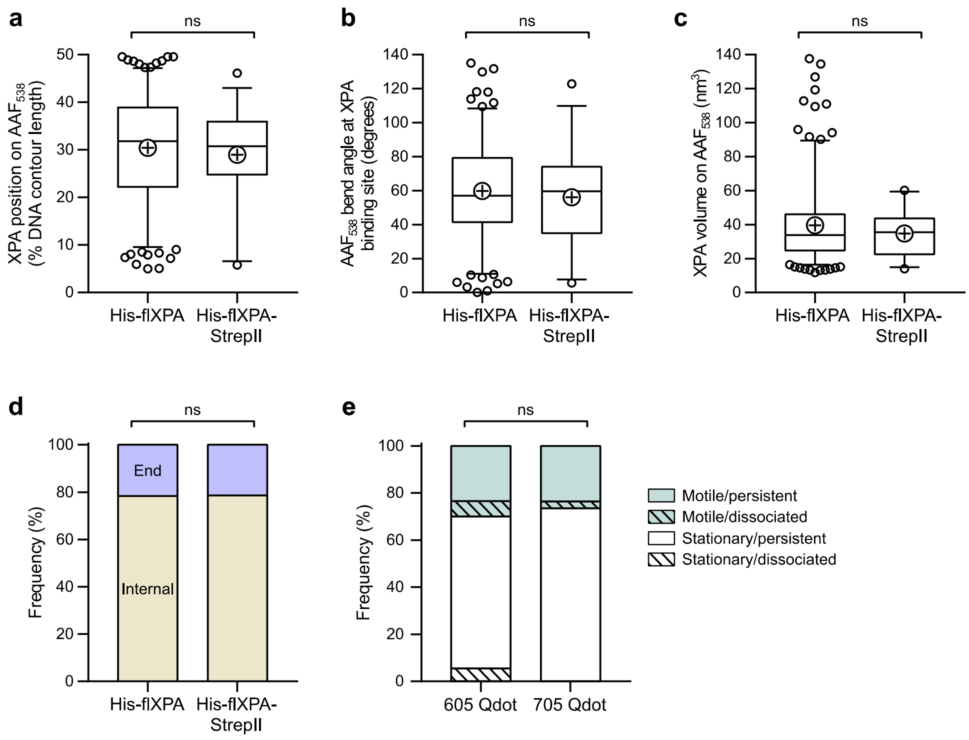

Supplementary Figure 5. Comparison of XPA preparations and Qdot labeling strategies. a, Box and whisker plot (5-95 percentile) showing binding positions on AAF_{538} of internally-bound His-flXPA (n = 217, data reproduced from Fig. 1e for comparison) and His-flXPA-StrepII (n = 33), determined by AFM. ns, p = 0.4810 by two-tailed Student's *t* test (p = 0.4628 by F test to compare variances). **b,** Box and whisker plot (5-95 percentile) showing DNA bend angles at all sites of internally bound XPA on AAF_{538}, determined by AFM. Results obtained with His-flXPA (n = 181, data reproduced from Fig. 4h for comparison) and His-flXPA-StrepII (n = 32) are shown. ns, p = 0.4996 by two-tailed Student's *t* test (p = 0.8013 by F test to compare variances). **c,** Box and whisker plot (5-95 percentile) showing AFM volumes of His-flXPA (n = 235, data reproduced from Fig. 3d) and His-flXPA-StrepII (n = 35) on AAF_{538}. ns, p = 0.2289 by two-tailed Student's *t* test (p = 0.0001 by F test to compare variances). **d,** Percentage of His-flXPA (n = 277, data reproduced from Fig. 1c for comparison) and His-flXPA-StrepII (n = 42) bound to DNA at ends (lavender) or internally (tan) on AAF_{538}, determined by AFM. ns, p = 0.9728 by χ^2 test. **e,** Stacked bar graph showing the fraction of motile (teal) vs. stationary (white) and persistent (solid) vs. dissociating (diagonal lines) Qdot-labeled His-flXPA particles on $UV\lambda_{20J}$ tightropes. Results obtained with 605 Qdot (n = 107) and 705 Qdot (n = 34) labeling strategies are shown (see Methods). Data reproduced as a sub-set of Fig. 5b. ns, p = 0.4214 by χ^2 test.

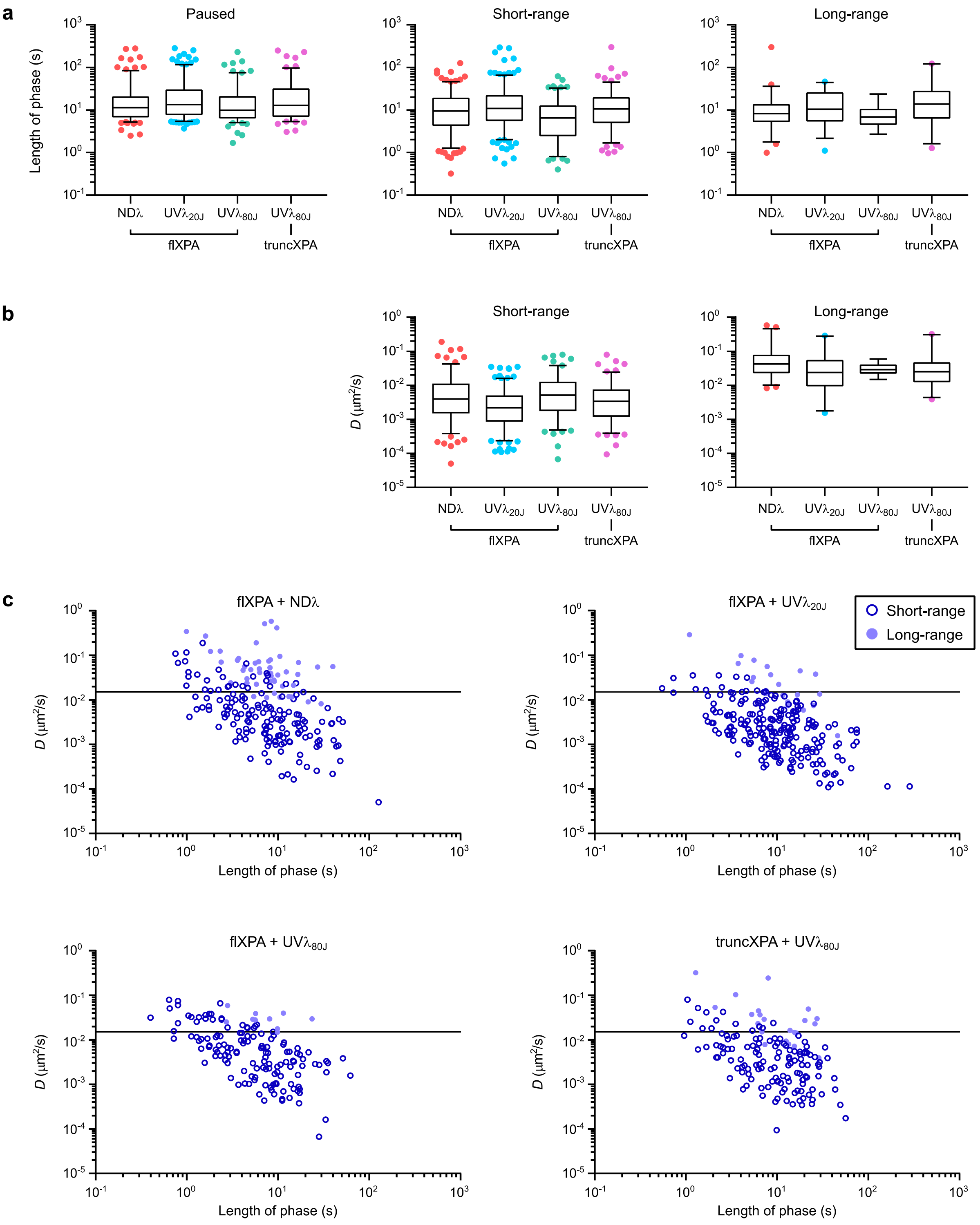

Supplementary Figure 6. Comparison of phase lengths and diffusion coefficients between experiments. a, Box and whisker plots (5-95 percentile) of the lengths (in seconds) of all measured phases for motile XPA particles. Left, paused mode: flXPA on NDλ (n = 193 phases), flXPA on $UV\lambda_{20J}$ (n = 239), flXPA on $UV\lambda_{80J}$ (n = 157), truncXPA on $UV\lambda_{80J}$ (n = 136). Center, short-range mode: flXPA on NDλ (n = 214), flXPA on $UV\lambda_{20J}$ (n = 254), flXPA on $UV\lambda_{80J}$ (n = 150), truncXPA on $UV\lambda_{80J}$ (n = 154).

Right, long-range mode: flXPA on NDλ (n = 55), flXPA on UVλ_{20J} (n = 27), flXPA on UVλ_{80J} (n = 10), truncXPA on UVλ_{80J} (n = 27). All comparisons within each mode by Dunn's multiple comparisons test were not significant ($p > 0.05$) except for: paused flXPA/NDλ vs. flXPA/UVλ_{20J} ($p = 0.0025$), short-range flXPA/NDλ vs. flXPA/UVλ_{80J} ($p = 0.0005$), short-range flXPA/UVλ_{20J} vs. flXPA/UVλ_{80J} ($p < 0.0001$), and short-range flXPA/UVλ_{80J} vs. truncXPA/UVλ_{80J} ($p < 0.0001$). Data reproduced from Fig. 6a, but separated to show variation between experimental conditions. **b,** Box and whisker plots (5-95 percentile) of *D* of all analyzed phases. Center, short-range mode: flXPA on NDλ (n = 156 phases), flXPA on UVλ_{20J} (n = 211), flXPA on UVλ_{80J} (n = 138), truncXPA on UVλ_{80J} (n = 131). Right, long-range mode: flXPA on NDλ (n = 49), flXPA on UVλ_{20J} (n = 20), flXPA on UVλ_{80J} (n = 10), truncXPA on UVλ_{80J} (n = 22). All comparisons within each mode by Dunn's multiple comparisons test were not significant ($p > 0.05$) except for: short-range flXPA/NDλ vs. flXPA/UVλ_{20J} ($p < 0.0001$) and short-range flXPA/UVλ_{20J} vs. flXPA/UVλ_{80J} ($p < 0.0001$). Data reproduced from Fig. 6b, but separated to show variation between experimental conditions. **c,** Plots of diffusion coefficient (*D*) vs. length of phase. Line drawn at D_{lim}, theoretical limit to *D* for free diffusion of Qdot-flXPA. flXPA on NDλ, n = 205 phases. flXPA on UVλ_{20J}, n = 231. flXPA on UVλ_{80J}, n = 148. truncXPA on UVλ_{80J}, n = 153. Data reproduced from Fig. 6c, but separated to show variation between experimental conditions.

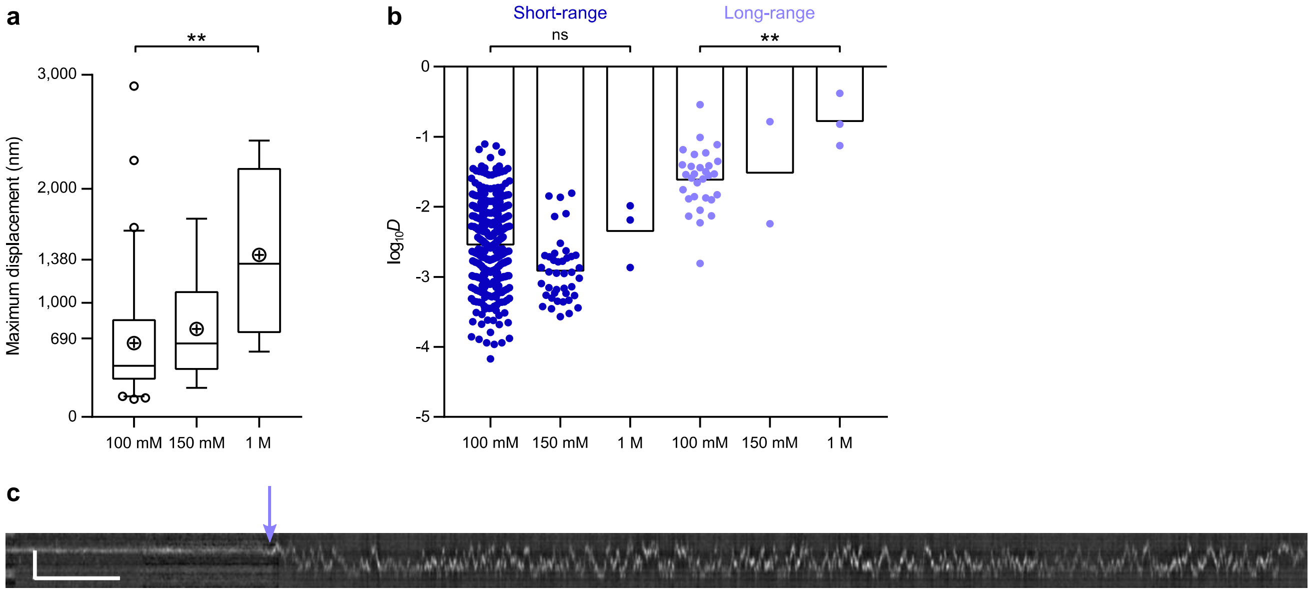

Supplementary Figure 7. Effect of ionic strength on XPA diffusion. a, Box and whisker plot (5-95 percentile) showing the maximum displacement of motile XPA particles on UVλ$_{20J}$ and UVλ$_{80J}$ with buffer containing 100 mM KCl (n = 61), 150 mM NaCl (n = 11), or 1 M NaCl (n = 4). ⊕, sample mean. ** p = 0.0045 by Post test for linear trend. **b**, Plot of the log transform of the diffusion coefficient (*D*) of motile XPA on UVλ$_{20J}$ and UVλ$_{80J}$ with buffer containing 100 mM KCl, 150 mM NaCl, or 1 M NaCl. The 100 mM data are reproduced from Fig. 5c (UVλ$_{20J}$ and UVλ$_{80J}$ only, subset of total), shown for comparison. Circles show individual data points, bars show means. ns, p = 0.5613; **, p = 0.0051 by Post test for linear trend. **c**, Example kymograph of flXPA on UVλ$_{20J}$. Starting buffer contains 100 mM KCl. The arrow indicates the transition from paused to long-range diffusion after the addition of 1 M NaCl. Scale bar, 2 µm (vertical) and 20 s (horizontal).

Supplementary References

1 Yang, Y., Sass, L. E., Du, C., Hsieh, P. & Erie, D. A. Determination of protein-DNA binding constants and specificities from statistical analyses of single molecules: MutS-DNA interactions. *Nucleic Acids Res* **33**, 4322-4334, doi:10.1093/nar/gki708 (2005).

2 Baldwin, M. R. & O'Brien, P. J. Nonspecific DNA binding and coordination of the first two steps of base excision repair. *Biochemistry* **49**, 7879-7891, doi:10.1021/bi100889r (2010).

3 Sawaya, M. R., Prasad, R., Wilson, S. H., Kraut, J. & Pelletier, H. Crystal structures of human DNA polymerase beta complexed with gapped and nicked DNA: evidence for an induced fit mechanism. *Biochemistry* **36**, 11205-11215, doi:10.1021/bi9703812 (1997).

4 Kong, M. *et al.* Single-Molecule Imaging Reveals that Rad4 Employs a Dynamic DNA Damage Recognition Process. *Mol Cell* **64**, 376-387, doi:10.1016/j.molcel.2016.09.005 (2016).

5 Kad, N. M., Wang, H., Kennedy, G. G., Warshaw, D. M. & Van Houten, B. Collaborative dynamic DNA scanning by nucleotide excision repair proteins investigated by single- molecule imaging of quantum-dot-labeled proteins. *Mol Cell* **37**, 702-713, doi:10.1016/j.molcel.2010.02.003 (2010).

6 Jones, C. J. & Wood, R. D. Preferential binding of the xeroderma pigmentosum group A complementing protein to damaged DNA. *Biochemistry* **32**, 12096-12104 (1993).

7 Arnspang, E. C., Brewer, J. R. & Lagerholm, B. C. Multi-color single particle tracking with quantum dots. *PLoS One* **7**, e48521, doi:10.1371/journal.pone.0048521 (2012).

8 Schurr, J. M. The one-dimensional diffusion coefficient of proteins absorbed on DNA. Hydrodynamic considerations. *Biophys Chem* **9**, 413-414 (1979).

9 Bagchi, B., Blainey, P. C. & Xie, X. S. Diffusion constant of a nonspecifically bound protein undergoing curvilinear motion along DNA. *J Phys Chem B* **112**, 6282-6284, doi:10.1021/jp077568f (2008).

10 Slutsky, M. & Mirny, L. A. Kinetics of protein-DNA interaction: facilitated target location in sequence-dependent potential. *Biophysical journal* **87**, 4021-4035, doi:10.1529/biophysj.104.050765 (2004).

11 Pei, J., Kim, B. H. & Grishin, N. V. PROMALS3D: a tool for multiple protein sequence and structure alignments. *Nucleic Acids Res* **36**, 2295-2300, doi:10.1093/nar/gkn072 (2008).

12 Romero, P. *et al.* Sequence complexity of disordered protein. *Proteins* **42**, 38-48 (2001).

13 Sugitani, N., Sivley, R. M., Perry, K. E., Capra, J. A. & Chazin, W. J. XPA: A key scaffold for human nucleotide excision repair. *DNA Repair (Amst)* **44**, 123-135, doi:10.1016/j.dnarep.2016.05.018 (2016).
