# Supplementary material for: Single molecule analysis reveals monomeric XPA bends DNA and undergoes episodic linear diffusion during damage search
Source: Nat Commun. 2020 Mar 13;11:1356. doi: 10.1038/s41467-020-15168-1 (PMC7069974; doi:10.1038/s41467-020-15168-1)
Supplement: Supplementary file 6 — Reporting Summary [file 41467_2020_15168_MOESM6_ESM.pdf]

nature research

Corresponding author(s): Bennett Van Houten

Last updated by author(s): Jan 20, 2020

Reporting Summary

Nature Research wishes to improve the reproducibility of the work that we publish. This form provides structure for consistency and transparency in reporting. For further information on Nature Research policies, see Authors & Referees and the Editorial Policy Checklist.

Statistics

For all statistical analyses, confirm that the following items are present in the figure legend, table legend, main text, or Methods section.

n/a	Confirmed	
☐	☒	The exact sample size (n) for each experimental group/condition, given as a discrete number and unit of measurement
☐	☒	A statement on whether measurements were taken from distinct samples or whether the same sample was measured repeatedly
☐	☒	The statistical test(s) used AND whether they are one- or two-sided<br>*Only common tests should be described solely by name; describe more complex techniques in the Methods section.*
☒	☐	A description of all covariates tested
☐	☒	A description of any assumptions or corrections, such as tests of normality and adjustment for multiple comparisons
☐	☒	A full description of the statistical parameters including central tendency (e.g. means) or other basic estimates (e.g. regression coefficient) AND variation (e.g. standard deviation) or associated estimates of uncertainty (e.g. confidence intervals)
☐	☒	For null hypothesis testing, the test statistic (e.g. F, t, r) with confidence intervals, effect sizes, degrees of freedom and P value noted<br>*Give P values as exact values whenever suitable.*
☒	☐	For Bayesian analysis, information on the choice of priors and Markov chain Monte Carlo settings
☒	☐	For hierarchical and complex designs, identification of the appropriate level for tests and full reporting of outcomes
☒	☐	Estimates of effect sizes (e.g. Cohen's d, Pearson's r), indicating how they were calculated

Our web collection on statistics for biologists contains articles on many of the points above.

Software and code

Policy information about availability of computer code

Data collection	All data collection described in Methods. AFM data collected via NanoScope software (Bruker). DNA tightrope data collected via NIS Elements AR (Nikon).
Data analysis	Data analysis described in Methods. DNA tightrope data were analyzed using ImageJ and MATLAB scripts (details, code, and sample data provided for reviewers). EMSA data were analyzed using ImageJ. All other analysis and plotting was done using GraphPad Prism.

For manuscripts utilizing custom algorithms or software that are central to the research but not yet described in published literature, software must be made available to editors/reviewers. We strongly encourage code deposition in a community repository (e.g. GitHub). See the Nature Research guidelines for submitting code & software for further information.

Data

Policy information about availability of data

All manuscripts must include a data availability statement. This statement should provide the following information, where applicable:

- Accession codes, unique identifiers, or web links for publicly available datasets
- A list of figures that have associated raw data
- A description of any restrictions on data availability

The data that support the findings of this study are available from the corresponding author upon reasonable request.

Field-specific reporting

Please select the one below that is the best fit for your research. If you are not sure, read the appropriate sections before making your selection.

☒ Life sciences ☐ Behavioural & social sciences ☐ Ecological, evolutionary & environmental sciences

Life sciences study design

All studies must disclose on these points even when the disclosure is negative.

Sample size	Sample sizes were determined according to commonly accepted standards in the field. For single molecule experiments, the sample size (n) is reported as number of particles (or other variable, as indicated) analyzed, from combined experiments. See below.
Data exclusions	n/a
Replication	Before combining experimental data obtained from different protein preparations or labeling strategies, data means and variances were compared by t test and F test or by chi-square (see Methods, Supp. Fig. 5, Supp. Fig. 6). Upon request, further detail about experimental replicates can be provided.
Randomization	n/a
Blinding	n/a

Reporting for specific materials, systems and methods

We require information from authors about some types of materials, experimental systems and methods used in many studies. Here, indicate whether each material, system or method listed is relevant to your study. If you are not sure if a list item applies to your research, read the appropriate section before selecting a response.

Materials & experimental systems

n/a	Involved in the study	
☐	☒	Antibodies
☒	☐	Eukaryotic cell lines
☒	☐	Palaeontology
☒	☐	Animals and other organisms
☒	☐	Human research participants
☒	☐	Clinical data

Methods

n/a	Involved in the study	
☒	☐	ChIP-seq
☒	☐	Flow cytometry
☒	☐	MRI-based neuroimaging

Antibodies

Antibodies used	All antibodies used in this study are commercially available from established vendors. Penta-His Biotin Conjugate, mouse IgG1 (Qiagen 34440). Penta-His Antibody, mouse IgG1 (Qiagen 34660).
Validation	Antibodies are validated by its manufacturers and cited in previous publications.
